# A Detention Hospital or Pavilion for Acute Mental Diseases1Read at the annual meeting of the Medical Society of the County of Onondaga, 1902.

**Published:** 1902-08

**Authors:** F. H. Stephenson

**Affiliations:** Syracuse, N. Y.; Neurologist and alienist to Syracuse Hospital for Women and Children, Syracuse Free Dispensary, Onondaga County and St. Vincent’s Orphanage, etc., 429 University Block.


### A Detention Hospital or Pavilion for Acute Mental Diseases.[1]

BY F. H. STEPHENSON, M. D., Syracuse, N. Y.,

Neurologist and alienist to Syracuse Hospital for Women and Children, Syracuse Free Dispensary, Onondaga County and St. Vincent's Orphanage, etc.

THIS is a subject of vital importance throughout both city and country, to both individuals and the public, relating to the appalling conditions which have always existed in the care of our insane patients, pending their commitment to some sanitarium or state hospital for the insane.

Locked in cells, like any common burglar or murderer. Just think of the situation! These people are mentally ill, without self control, or judgment. They are often placed in cells of the municipal lodging house, the court house, or possibly the penitentiary, there to await their examination, commitment, and arrival of attendants who are to take them to some sanitarium. Many of these people are physically ill, and their mental disturbance only temporary, passing off after a few days or few weeks of treatment.

1. Read at the annual meeting of the Medical Society of the County of Onondaga, 1902.

Another question presents itself: Should these people be at once adjudged insane, and sent to an asylum, forever injuring their prospects in life in many ways, especially so far as ever holding any important position? We answer, most assuredly not. Our own county should provide a small, suitable pavilion, in connection with one of our city hospitals, as has recently been done in Albany, for instance, and as many other cities and counties are now doing, where these cases can be observed and treated by physicians experienced in mental diseases. Then, when suitable time has been allowed for mental disturbance to subside, or it becomes evident that the patient's illness will be necessarily prolonged, requiring special treatment and management, then a council of physicians may commit such patients to whatever institution the family, friends and the county judge deem best.

I have, in my own practice, taken many patients through attacks of mania, melancholia and other acute mental derangements whose condition has been known only to intimate family friends. Some of these patients have married, others have resumed responsible and lucrative positions which could not have been possible had they been publicly declared insane, and committed to institutions for that class of people.

Many of you must have met similar cases in your own practice, which have mentally cleared after a few days, or a few weeks' treatment. Do not misunderstand me in this matter, thinking I disparage our noble state institutions for the insane. Far from it! We must have them. They are doing a great work, curing a larger percentage of the insane every year.

I have spent weeks in the asylums of this country, and Europe, and have no criticisms to offer. The medical care is scientific, the nursing and management most excellent, and the food all that is required. Sanitation, food and surroundings are often far superior to anything the great majority of such patients ever before enjoyed. I only insist that we properly care for these patients preparatory to entering these institutions, and keep them under proper observation for a suitable length of time to determine, if commitment be necessary or wise.

My friend, Dr. Frederick Peterson, president of the New York state board of lunacy, is very desirous that every city or county should be equipped in this respect and is bending every energy in this direction of improving the care of the insane. The time will certainly come when more humane consideration will be given these unfortunates. And can there be a better time than the present for pushing this subject before our humanitarians, politicians and the general public?

By way of illustration, may I ask your indulgence while I briefly report several cases which have come under my care, some within a few months, patients who fortunately were so circumstanced that they could receive special attention either in their own homes or in one of our city hospitals.

Case I.—Miss Q., aged 26 years, suffered from acute melancholia with terrifying delusions of persecution with depression, insomnia and incoherence of thought and expression. Patient tore her clothing, attempted suicide and would take food only under compulsion. On examination I found her anemic, thin, pale, tongue coated, constipated, urine scanty, loaded with urates, epithelium and pus; no albumen, sugar or casts; specific gravity heavy; urea scarcely $\frac{1}{2}$ per cent. Had not slept for ten nights. Under treatment this patient made a perfect recovery, home care having been all that was necessary. She reëntered society, has married, has had no recurrence of the malady, and her illness is now only spoken of as some serious nervous trouble that for a time disturbed her mind.

Case II.—Miss B., was ill four months with acute mania with marked excitement and exhaustion. This case was dependent upon anemia; forced feeding, tonics, sedatives, rest, eliminatives, quiet, massage, and the like, brought about a condition of normal mentality and physical health. She was not removed from her home.

Case III.—Miss D., recently seen with Dr. Wynkoop, was excited, confused, incoherent, sleepless and delirious. A few days' rest, quiet, stuffing diet and tonics, with proper sedatives, brought about mental balance, and she convalesced quite rapidly from a condition of nervous exhaustion, dependent upon anemia, at the Woman's and Children's Hospital.

In such a case as this how evident is the wisdom and desirability of treating an unbalanced patient in one of our own hospitals rather than subjecting her to the publicity of commitment to an insane asylum in the beginning.

Case IV.—Mr. E., suffered from confusional insanity, insomnia and excitement due to an alcoholic debauch. A nurse, with the patient's family, cared for him until within four weeks. He made a good recovery without having been sent from home.

Case V.—Mrs. P., referred to me by Dr. Slingerland; at the menopause had an attack of melancholia, with confusion, delirium, insomnia, and marked prostration. Eight weeks in the Woman's and Children's Hospital, with special treatment and management, have resulted in a complete cure. Her husband told me recently he fully expected she would have to be sent to an asylum.

Case VI.—Miss F., a teacher in one of the public schools of Denver became overworked, developing neurasthenic conditions. Then followed the loss of all her savings in a wild cat investment. This produced a period of excitement, accompanied with violence, smashing of windows, and the like. After a few days of excitement, she settled into a condition of melancholia with delusions, but was quiet.

This patient made a perfect recovery in the Woman's and Children's Hospital, and had no difficulty in obtaining a good position in the high school of one of our neighboring towns. Had her nervous and mental condition been treated in an asylum, that fact alone would have prevented her securing so responsible a position in any place where the truth was known.

Case VII.—Dr. P., from a town not 500 miles from Syracuse, came to our city to consult a laryngologist for throat treatment, having a delusion that he had swallowed pieces of bone, that still remained in his throat. A second physician was consulted because the first did not remove them. He was then referred to me. Calling at his hotel, I learned that he was addicted to the use of cocaine, heroin and morphine. I arranged for his entrance into St. Joseph's Hospital on Monday, the following day, but a sudden impulse decided him to go at once to Albany; therefore he rushed to the train, getting on to the rear platform of the last car, his wife being unable to restrain him. While in Albany he was taken to the City Hospital. This was before the establishment of the detention pavilion. He made so much disturbance that he was arrested, taken to the lock-up, and came before the judge. When his mental condition was recognised, physicians were appointed to commit him to an asylum, where a few weeks' treatment sufficed to restore his mental equilibrium, and he resumed practice.

Imagine the anxiety and suffering which the doctor's wife and friends endured while he was thus shifting from place to place, being arrested, and the like. How they suffered from the notoriety and deprecated the injury to his practice. A hospital in our city, with a detention pavilion for acute cases, or mental suspects, would have prevented all this, and it must appeal to all of us as the only humane manner in which these patients can be cared for.

Mental disturbances are very frequently observed in people of neurotic temperament and ancestry, caused, or augmented by anemia, overwork, worry, or excess; also by the menopause, malassimilation, shock, debility, drug habits, and age. Often these mental symptoms are of brief duration, and a detention pavilion affords every facility that the general hospital and the special hospital combined can offer for the cure of these patients. Their ultimate recovery is not retarded by being transferred a few days' or weeks later, should continuance of mental disturbance decide in favor of so doing.

Of course we all realise the strong probability of a recurrence of many of these mental maladies, and that a mental scar is liable to follow insanity. But a sick and unbalanced brain is no disgrace, and is as real an illness as a disordered liver, stomach, appendix, lung, or any other organic or functional disease.

But we cannot disguise the fact that a mental malady in the eye of the world is a stain upon a family, and we physicians are placed where we should and must protect the masses-not only by instruction in correct living—how to keep mentally and physically well—but it is also our duty to ensure prompt and thorough care of minds diseased, protecting such unfortunates and their friends from unnecessary publicity, mental anxiety, oversensitiveness and injury to their social and business standing. We can only accomplish this by providing, as soon as may be, a suitable and satisfactory retreat, which will prevent a premature and needless commitment and detention in the regular hospitals for the insane.

429 University Block.

